# Interactive effect of social isolation and poor nutritional status on cognitive function decline in older adults

**DOI:** 10.3389/fnut.2026.1744184

**Published:** 2026-04-08

**Authors:** Lian Li, Sa Xiao, Zile Zhang, Hongying Yang, Yumin Tao

**Affiliations:** 1 Department of Psychiatry, Zhejiang Key Laboratory of Drug Addiction and Brain Health, Affiliated Kangning Hospital of Ningbo University, Ningbo, Zhejiang, China; 2 Department of Psychiatry, Ningbo Kangning Hospital, Ningbo, Zhejiang, China; 3 School of Public Health, Ningbo University Health Science Center, Ningbo University, Ningbo, Zhejiang, China; 4 School of Mathematical Sciences, Zhejiang University, Hangzhou, Zhejiang, China

**Keywords:** cognitive decline, interactive effect, olderadults, poor nutritional status, social isolation

## Abstract

**Background:**

Social isolation and poor nutritional status are well-established independent risk factors for cognitive decline. However, few studies have examined their combined effects on cognitive decline. This cross-sectional study evaluated the synergistic association between social isolation, poor nutritional status, and cognitive decline in older adults aged 60 years and above.

**Methods:**

Data from 10,501 older Chinese adults were analyzed. Univariate and multivariate logistic regression analyses were conducted to determine the relationship between social isolation, poor nutritional status, and cognitive decline. Interaction effects were evaluated using the relative excess risk due to interaction (RERI), attributable proportion due to interaction (AP), and synergy index (S).

**Results:**

The prevalence of cognitive decline in older adults was 12.87%. Multivariate analysis revealed odds ratios for cognitive decline associated with social isolation and poor nutritional status of 1.580 [95% confidence interval (CI): 1.379–1.811] and 3.667 (95% CI: 3.200–4.203), respectively. A significant synergistic interaction effect between social isolation and poor nutritional status on cognitive decline was observed (RERI = 2.199, AP = 0.362, S = 1.765), and this effect persisted across sex and age subgroups.

**Conclusion:**

Among older Chinese adults, social isolation and poor nutritional status exert a significant synergistic association with cognitive decline. These findings highlight the importance of integrated interventions targeting both social isolation and nutritional status to support cognitive health in older populations.

## Introduction

Most countries worldwide are experiencing rapid population aging. By 2050, individuals aged 60 years and above are projected to comprise 22% of the global population (1). China, which has the largest older population, is expected to have more than 500 million individuals aged 60 years and above by 2048 (2). With this demographic shift, age-related conditions such as cognitive decline and dementia have become critical public health challenges (3). In China, an estimated 38.77 million and 15.07 million adults aged 60 years and above experience cognitive decline and dementia, respectively (4, 5). These conditions are the leading causes of disability among older adults, imposing substantial health and economic burdens.

Social isolation, defined as living alone or having limited social interactions with friends, relatives, or others, is associated with cognitive decline (6). According to the Dementia Prevention, Intervention, and Care 2024 report, reducing social isolation could lower dementia incidence by 5% (7). Two Chinese cohort studies have reported an association of high baseline social isolation with subsequent cognitive decline (8, 9). Older adults also have an increased risk of poor nutritional status (10), which exacerbates age-related diseases. Studies have indicated that poor nutritional status is associated with cognitive decline in Chinese older adults, and malnourished individuals are more likely to experience poor cognitive outcomes (11, 12). Studies also have suggested a bidirectional relationship between social isolation and poor nutritional status in older adults (13, 14). Both conditions are independently associated with cognitive decline (15, 16). However, few studies have investigated their combined impact on cognitive decline.

This study examined the association between social isolation, poor nutritional status, and the risk of cognitive decline. We hypothesized that individuals experiencing both social isolation and poor nutritional status have a higher risk of cognitive decline than those experiencing either condition alone and that these two factors interact synergistically. To determine the independent effects of social isolation and poor nutritional status, we controlled for potential confounders, namely age, sex, education level, marital status, alcohol consumption, smoking status, hearing loss, chronic disease status, depression, and loneliness.

## Methods

### Study design and population

Ningbo, an economically developed city with six districts and four counties, had a population of approximately 9.7 million in 2024, of whom 18.56% were aged 60 years and above. We conducted a cross-sectional study among adults aged 60 years and above in Ningbo from April to December 2024. Participants were selected using a multistage random cluster sampling method. First, three districts and two counties were randomly chosen. Second, one town or street was randomly selected from each district or county. Third, one community or village was randomly selected from each town or street. Finally, eligible older adults from the selected communities or villages were included in the study. The inclusion criteria were (1) residing in Ningbo for more than a year, (2) an age of ≥60 years, (3) participating voluntarily and demonstrating the ability to complete the questionnaire, and (4) having no diagnosis of Alzheimer’s disease or other types of dementia. The exclusion criteria were (1) not signing the consent form and (2) missing key variables required in this study. Participants completed the questionnaires electronically. A total of 10,530 individuals were initially approached to participate in the survey, and 29 participants were excluded due to being under 60 years of age. Finally, 10,501 participants who had provided written or electronic informed consent and had complete data for all key variables (including BSSD, social isolation items, MNA-SF, and other covariates) were included in our analysis. All investigators underwent a standardized training program prior to initiating the formal data collection. This program comprehensively covered the theoretical foundations and practical application of the assessment instruments, including scale structure, administration protocols, scoring guidelines, and standardized interview techniques-thereby ensuring procedural consistency and inter-rater reliability.

### Cognitive function assessment

Global cognitive function was assessed using the Chinese version of Brief screening Scale for Dementia (BSSD) (17). The BSSD is a brief cognitive screening tool with high sensitivity and specificity and consists of eight cognitive domains: common sense and image understanding (6 points), short-term memory (3 points), instant memory (3 points), attention and calculation (3 points), location targeting (5 points), time targeting (4 points), language (3 points) and naming ability (3 points). The total scores range from 0 to 30, with higher scores indicating better global cognitive function. The cutoffs for the BSSD used in the current study were ≤18 points for illiterate individuals, ≤21 points for those with an elementary school education, and ≤24 points for those with a middle school education or higher (17).

### Social isolation and nutritional status assessment

Social isolation was defined using three questions: “Do you live with other people?”; a response of “No” was scored 1, “How many times a month do you visit a friend or family member?”; a response of “Visited less than once/month” was scored 1, and “How many times a month do you participate in social activities”; a response of “Participated less than once per week” was scored 1. A total score of 2 or 3 indicated social isolation, whereas a score of 0 or 1 indicated no social isolation (18, 19). Nutritional status was evaluated using the Mini Nutritional Assessment Short Form (MNA-SF) (20), a validated, sensitive, and rapid screening tool with performance comparable to that of the full Mini Nutritional Assessment. The MNA-SF examines six items: recent appetite loss (2 points), recent weight loss (3 points), mobility (2 points), stress or acute disease (2 points), neuropsychological problems (2 points), and body mass index (3 points). In this study, MNA-SF scores below 8 indicated malnutrition, scores between 8 and 11 indicated a risk of malnutrition, and scores above 11 indicated a well-nourished state (21). For analysis, we grouped individuals classified as malnourished or at risk of malnutrition into a single “poor nutritional” category.

### Covariate assessment

We examined sociodemographic characteristics, namely age (continuous), sex (male or female), residence location (urban or rural), education level (<6 or ≥6 years), marital status (married or other), alcohol consumption (yes or no), smoking (yes or no), hearing loss (yes or no), presence of chronic noncommunicable diseases (yes or no), and loneliness (yes or no). Chronic noncommunicable diseases and hearing loss were self-reported or clinically diagnosed. Loneliness was measured using using a single-item self-report question: “How many times do you feel lonely in the past month?”, with responses of “sometimes”, “often” and “always” were classified as experiencing loneliness. Depression was assessed using the 9-item Patient Health Questionnaire, and scores of ≥10 indicated the presence of depressive symptoms (22).

### Statistical analysis

Differences in quantitative variables with a normal distribution were examined using the *t* test and are reported as means ± standard deviations. Differences in categorical variables were analyzed using the chi-square test and are presented as numbers (percentages). After adjusting for confounding factors, we conducted logistic regression analysis to examine the association between social isolation, poor nutritional status, and cognitive decline. Sex and age may act as effect modifiers on the associations of social isolation and poor nutritional status with cognitive decline among older adults. We therefore performed sex- and age-stratified analyses to assess the consistency and robustness of the observed interactive effects. Additive interaction effects were calculated using an Excel spreadsheet provided in a previous study (23). Additive interaction is defined as a departure from the expected additive effects of two exposures, which is considered more meaningful for public health and clinical applications than multiplicative interaction. Additive interaction effects were evaluated using the relative excess risk due to interaction (RERI), attributable proportion due to interaction (AP), and synergy index (S), and their 95% confidence interval (CIs) and *p* values were calculated using the delta method (24, 25). An additive interaction was considered present if the 95% CI of RERI was >0, the 95% CI of AP was >0, or the 95% CI of S was >1 (25). A *p* value of <0.05 was considered statistically significant. All statistical analyses were conducted using SPSS 22.0 (IBM Corp., Armonk, NY, USA).

## Results

A total of 10,501 participants were recruited, of whom 4,652 (44.30%) were men. Participants with cognitive decline (case group) were significantly older than those without cognitive decline (control group; 75.60 ± 6.78 vs. 72.01 ± 5.72 years, *p* < 0.001; Table 1). Compared with the case group, the control group had higher proportions of men, living in rural, lower education year, drinkers, and smokers. By contrast, the proportions of participants with hearing loss, chronic diseases, depression and loneliness were higher in the case group than in the control group (Table 1).

**Table 1:** Baseline characteristics of participants.

Characteristics		Without cognitive decline	With cognitive decline	*t/x^2^*	*P*
Age		72.01 ± 5.72	75.60 ± 6.78	−18.559	<0.001
Sex	Men	4,099 (44.8)	553 (40.9)	7.262	0.007
	Women	5,050 (55.2)	799 (59.1)		
Residence location	Urban	6,160 (67.3)	1,025 (75.8)	39.240	<0.001
	Rural	2,989 (32.7)	327 (24.2)		
Education year	<6 years	7,097 (77.6)	889 (65.8)	90.307	<0.001
	≥6 years	2052 (22.4)	463 (34.2)		
Marital status	Married	8,196(89.6)	1,105 (81.7)	71.766	<0.001
	Others	953 (10.4)	247 (18.3)		
Drinking	Yes	1,363 (14.9)	160 (11.8)	8.915	0.003
	No	7,786 (85.1)	1,192 (88.2)		
Smoking	Yes	1966 (21.5)	250 (18.5)	6.357	0.012
	No	7,183(78.5)	1,102 (81.5)		
Hearing loss	Yes	666 (7.3)	237 (17.5)	157.459	<0.001
	No	8,483 (92.7)	1,115 (82.5)		
Chronic diseases	Yes	5,919 (64.7)	958 (70.9)	19.782	<0.001
	No	3,230 (35.3)	394 (29.1)		
Depression	Yes	26 (0.3)	48 (3.6)	179.578	<0.001
	No	9,123 (99.7)	1,304 (96.4)		
Loneliness	Yes	963 (10.5)	262 (19.4)	89.590	<0.001
	No	8,186 (89.5)	1,090 (80.6)		
Social isolation	Yes	2,681 (29.3)	626 (46.3)	157.751	<0.001
	No	6,468 (70.7)	726 (53.7)		
Nutritional status	Well nourished	7,924 (86.6)	808 (59.8)	623.722	<0.001
	At risk	1,209 (13.2)	525 (38.8)		
	Malnourished	16 (0.2)	19 (1.6)		

A total of 1,352 participants were classified as having cognitive decline, accounting for 12.87% of the total sample. The overall prevalence of social isolation and poor nutritional status was 31.49 and 16.85%, respectively. Compared with the control group, the case group had a significantly higher prevalence of social isolation (46.3% vs. 29.3%) and poor nutritional status (40.2% vs. 13.4%; *p* < 0.001 for both; Table 1 and Supplementary Table 1).

Table 2 presents the results of univariate and multivariate logistic regression examining the association between social isolation, poor nutritional status, and cognitive decline. The results indicated a strong association of social isolation and poor nutritional status with cognitive decline. After adjusting for multiple variables, we observed that older Chinese adults who were socially isolated were 1.580 times more likely to experience cognitive decline than those who were not socially isolated (odds ratio [OR]: 1.580, 95% CI: 1.379–1.811, Table 2). In addition, poor nutritional status was associated with an increased likelihood of cognitive decline in older adults (OR: 3.667, 95% CI: 3.200–4.203, Table 2). The variance inflation factor (VIF) values of the variables in the multivariate logistic regression model ranged from 1.035 to 1.523. This significant association remained consistent in sex and age subgroup analyses (Table 3).

**Table 2:** The association between social isolation and poor nutritional status on the risk of cognitive decline in the older adults.

Characteristics	OR (95% CI)	
	Model 1	Model 2
Social isolation	1.957 (1.736–2.205)	1.580 (1.379–1.811)
Poor nutritional status	4.199 (3.700–4.760)	3.667 (3.200–4.203)

The model 1 was unadjusted for confounding factors. The model 2 was adjusted for age, sex, residence location, education year, marital status, drinking status, smoking status, hearing loss, chronic disease status, depression, loneliness, nutritional status or social isolation. Poor nutritional status including both malnourished and at risk of malnutrition.

**Table 3:** The association between social isolation and poor nutritional status on the risk of cognitive decline in the sex and age subgroups.

Subgroups	OR (95% CI)	
Sex	Social isolation	Poor nutritional status
Men	1.919 (1.562–2.356)	3.225 (2.605–3.991)
Women	1.362 (1.133–1.636)	4.044 (3.382–4.835)
Age		
<75 years	1.533 (1.262–1.863)	3.691 (3.034–4.490)
≥75 years	1.605 (1.324–1.946)	3.679 (3.040–4.452)

OR was adjusted for the same covariates as model 2 in Table 2.

Table 4 presents the interaction effects between social isolation and poor nutritional status on cognitive decline. Compared with participants without social isolation and poor nutritional status, those experiencing both conditions had 6.071 times higher odds of cognitive decline (OR = 6.071, 95% CI: 4.942–7.458). Social isolation and poor nutritional status had significant positive additive interaction effects on cognitive decline (RERI = 2.199, AP = 0.362, S = 1.765). These significant additive interaction effects were also observed in sex and age subgroup analyses (Tables 5, 6).

**Table 4:** The interactive items between social isolation and poor nutritional status on the risk of cognitive decline in the older adults.

Social isolation	Nutritional status	*N*	OR (95% CI)	RERI	AP	*S*
No	Good	6,107	1	2.199 (1.043–3.356)	0.362 (0.225–0.500)	1.765 (1.343–2.320)
No	Poor	1,087	3.388 (2.833–4.052)			
Yes	Good	2,625	1.488 (1.264–1.751)			
Yes	Poor	682	6.071 (4.942–7.458)			

OR was adjusted for the same covariates as model 2 in Table 2. RERI, relative excess risk of interaction; AP, attributable proportion due to interaction; S, synergy index.

**Table 5:** The interactive items between social isolation and poor nutritional status on the risk of cognitive decline in the sex subgroup.

Sex	Social isolation	Nutritional status	OR	RERI	AP	S
Men	No	Good	1	2.713 (0.727–4.698)	0.416 (0.217–0.614)	1.964 (1.284–3.004)
	No	Poor	3.000 (2.282–3.943)			
	Yes	Good	1.817 (1.426–2.315)			
	Yes	Poor	6.529 (4.712–9.049)			
Women	No	Good	1	1.739 (0.244–3.233)	0.303 (0.098–0.508)	1.580 (1.087–2.296)
	No	Poor	3.722 (2.933–4.723)			
	Yes	Good	1.278 (1.025–1.592)			
	Yes	Poor	5.739 (4.393–7.499)			

OR was adjusted for the same covariates as model 2 in Table 2.

**Table 6:** The interactive items between social isolation and poor nutritional status on the risk of cognitive decline in the age subgroup.

Age	Social isolation	Nutritional status	OR	RERI	AP	S
<75 years	No	Good	1	2.786 (0.757–4.816)	0.416 (0.217–0.614)	1.956 (1.283–2.980)
	No	Poor	3.417 (2.683–4.351)			
	Yes	Good	1.499 (1.195–1.879)			
	Yes	Poor	6.702 (4.927–9.117)			
≥75 years	No	Good	1	2.326 (0.642–4.010)	0.349 (0.161–0.538)	1.698 (1.184–2.433)
	No	Poor	3.713 (2.848–4.840)			
	Yes	Good	1.622 (1.283–2.052)			
	Yes	Poor	6.661 (5.052–8.782)			

OR was adjusted for the same covariates as model 2 in Table 2.

## Discussion

This is the first large-scale cross-sectional study to examine the interactive effects of social isolation and poor nutritional status on cognitive decline among community-dwelling older adults. The prevalence of cognitive decline in the present study was 12.87%. The findings indicate that social isolation and poor nutritional status were independently associated with cognitive decline. Moreover, the presence of both social isolation and poor nutritional status exerted a synergistic interaction effect on the risk of cognitive decline. Specifically, 2.199 of the relative excess risk was attributed to the additive interaction between social isolation and poor nutritional status.

Social isolation can contribute to cognitive decline. A meta-analysis of 51 studies reported that older adults with larger social networks and greater engagement in social activities had better cognitive function later in life than those with smaller networks and lower engagement (*r* = 0.054, *p* < 0.05) (26). Similarly, the present study revealed that social isolation was significantly associated with cognitive decline, and this association appeared to be independent of loneliness. Several theories have been proposed to explain the association between social isolation and cognitive function. The first theory, known as the stress-buffering hypothesis, suggests that strong social support helps mitigate the effects of daily stressful events and stress responses (27). However, individuals who are socially isolated lack adequate support, and stress and stressors can hyperactivate the hypothalamic–pituitary–adrenal axis and release more glucocorticoids, which may lead to hippocampal neurodegeneration (28). The second theory posits that social isolation itself acts as a chronic stressor and reduces connectivity and plasticity in the prefrontal cortex, eventually impairing cognitive function (29, 30). The third theory, known as the cognitive reserve hypothesis, suggests that greater cognitive stimulation through social activities enhances cognitive reserve, thereby supporting cognitive function (15, 31). In addition, social isolation is associated with an increased risk of vascular diseases (32), which, in turn, can contribute to cognitive decline (33).

Our study supports previous evidence that poor nutritional status is associated with a decline in cognitive function. Leirós et al. reported that individuals with poorer nutritional status, mainly assessed using the MNA-SF, were more likely to have lower cognitive function (34). Furthermore, in Chinese oldest-old and centenarian adults, malnutrition was found to be positively associated with cognitive decline even after adjusting for potential confounders (12). Previous studies (11, 35) conducted in China have identified malnutrition as a key factor correlating with cognitive decline. The relationship between poor nutritional status and cognitive decline is complex and reciprocal. Poor nutritional status may contribute to synaptic dysfunction, promote neuronal loss, and lead to cerebral cortex thinning, ultimately resulting in cognitive decline (11, 36). Other studies have found that poor nutritional status, characterized by inadequate protein and nutrient intake, can lead to insufficient neuronal energy, nerve cell damage, and central nervous system deregulation, ultimately resulting in the development of neurological diseases (12, 37). In addition, older adults with cognitive decline may have an impaired cognitive ability to manage dietary intake effectively, leading to decreased nutrient intake and worsening nutritional status.

A critical finding of the present study is the additive effect of social isolation and poor nutritional status on cognitive decline, and this effect persisted across sex and age subgroups. Although both factors were independently associated with cognitive decline, their co-occurrence significantly increased this risk, and 36.2% of the combined effect was likely to be attributable to their interaction. Socially isolated individuals are more likely to adopt poor dietary habits because of decreased access to shared meals and a lack of motivation to prepare nutritious food (14, 38). Conversely, poor nutritional status can lead to physical frailty and decreased mobility (39), further limiting social engagement and perpetuating a cycle of isolation and cognitive decline (40). Previous studies have not identified an additive effect of social isolation and poor nutritional status. Therefore, the specific mechanism of their interaction remains unclear. A possible explanation is that both factors contribute to the release of proinflammatory cytokines that regulate neuroinflammation and, in turn, affect the function and structure of the cerebral cortex (29, 30, 37). The co-occurrence may exacerbate these effects, further impairing cortical function. Future studies should explore these pathways in greater depth using longitudinal designs and biomarkers to determine temporal relationships between these risk factors and cognitive outcomes.

Routine geriatric assessments should include screening for both social isolation and poor nutritional status because these are modifiable risk factors for cognitive decline that can be addressed through targeted interventions. Expanding social networks and implementing effective nutritional interventions for high-risk older adults may help reduce the risk of cognitive dysfunction. Community-based programs that promote social engagement, such as group activities and intergenerational initiatives, could help mitigate the effects of social isolation. Concurrently, nutritional support, including the mitigation of food insecurity (41), personalized dietary guidance (such as adherence to the Mediterranean diet) should be integrated into care strategies for older adults at poor nutritional status (42). Public health efforts should emphasize the dual importance of social connectivity and proper nutrition in preserving cognitive function.

## Strengths and limitations

This study has several strengths. First, our survey included a large sample size, enhancing the reliability of the data. Second, our study focused on a key population, that is, older adults. Finally, we investigated the synergistic effects of social isolation and poor nutritional status and identified valuable findings. However, this study has some limitations that should be addressed. First, this study used a multistage cluster sampling design. However, sampling weights and cluster identifiers were not available; thus, we were unable to adjust for clustering effects in the statistical analyses, which may lead to slightly narrower confidence intervals. Second, as this was a cross-sectional study, causal inferences cannot be drawn. Reverse causality cannot be excluded, as cognitive decline may lead to decreased social interaction and subsequent exacerbation of social isolation. Future studies should use longitudinal designs to determine temporal relationships and incorporate objective measures, such as biomarkers of nutritional status and electronic tracking of social interactions. Third, the use of self-reported questionnaires may introduce nonresponse bias and self-reporting inaccuracies, potentially affecting the validity of the findings. Given that participants were required to complete the questionnaire, this method could have excluded older adults with the most severe impairments, making some degree of selection bias inevitable. Loneliness and depression are both recognized as risk factors for cognitive decline (15, 43). Furthermore, existing research has indicated that social isolation is associated with loneliness and depression (18, 19), which may increase the risk of over-adjustment when loneliness and depression are included as covariates in the analysis. In addition, in the absence of a clinical diagnosis or comprehensive cognitive testing, the measures of cognitive function used in this study may not be sensitive enough to detect early cognitive impairment.

## Conclusion

The results of this study indicate that social isolation and poor nutritional status are positively associated with cognitive decline in older Chinese adults. Furthermore, co-occurrence of these two factors is associated with an excess odds of cognitive decline. Our findings suggest that interventions designed to preserve cognitive health must address both social and nutritional factors simultaneously to achieve optimal outcomes. Greater attention should be given to the social isolation and poor nutritional status of this population, including implementing community-based social interventions and providing timely nutritional support. By targeting these modifiable determinants, healthcare providers and policymakers can develop more effective strategies to promote cognitive health and reduce the burden of cognitive decline in aging populations.

## Data Availability

The original contributions presented in the study are included in the article/Supplementary material, further inquiries can be directed to the corresponding author.
