# Supplementary material for: Interactive effect of social isolation and poor nutritional status on cognitive function decline in older adults
Source: Front Nutr. 2026 Apr 8;13:1744184. doi: 10.3389/fnut.2026.1744184 (PMC13099300; doi:10.3389/fnut.2026.1744184)
Supplement: Supplementary file 1 [file Table_1.DOC]

Supplementary Table 1 The distributions of social isolation and poor nutritional status

|  | Social isolation | Poor nutritional status |
| --- | --- | --- |
| **All** | 3307 (31.5) | 1769 (16.8) |
| **Cognitive decline** |  |  |
| Yes | 626 (46.3) | 544 (40.2) |
| No | 2681 (29.3) | 1225 (13.4) |
| **Sex** |  |  |
| Men | 1343 (28.9) | 761 (16.4) |
| Women | 1964 (33.6) | 1008 (17.2) |
| **Age** |  |  |
| <75 years | 1861 (26.4) | 952 (13.5) |
| ≥75 years | 1446 (41.7) | 817 (23.6) |
